# 257 Chlorine Dioxide Versus Automated Vaporized Hydrogen Peroxide for High-Level Disinfection of Ultrasound Probes in Outpatient Practice

**DOI:** 10.1017/ash.2026.10624

**Published:** 2026-06-23

**Authors:** Jacob Pierce, Belk Kelsey

**Affiliations:** 1 Brody School of Medicine at East Carolina University

## Abstract

**Background:** Hand hygiene is the most important tool for preventing the spread of infections in the healthcare environment. Personal protective equipment (PPE) and nails policy are important additional measures for limiting infectious spread. Our facility typically performs both covert and overt hand hygiene, PPE and nail audits. Compliance rates with hand hygiene and PPE have declined since the COVID-19 pandemic. More information was needed on factors contributing to decreasing compliance rates. Methods An anonymous hybrid electronic and print survey was distributed at annual Infection Prevention fair across a 9-hospital system in Eastern North Carolina from April 2025-November 2025. The survey was conducted via Qualtrics. Results There were 432 total responses, 41% were registered nurses, 7% radiation technologists, 6% nursing assistants and 3% or less of a wide variety of other specialties/departments. Community hospitals represented 83% of responses and 17% were from a large academic medical center. Overall self-reported hand hygiene and PPE compliance was 96.10% and 94.3% whereas perceived compliance of peers was 89.29% and 88.7% respectively. Individual unit audits reported a rate of 89.9% hand hygiene compliance during this time period. When asked about specific scenarios, 84.6% of healthcare workers stated they would wear PPE for isolation precautions when entering the room, but patient contact was not anticipated. Conclusions Healthcare workers report high levels of compliance with hand hygiene and PPE but report lower numbers for perceived compliance of peers. In our health system opportunities to improve isolation precaution compliance include increasing availability of PPE and education on need for PPE even when patient contact is not anticipated. There is strong support for a natural nails policy among healthcare workers in our system, but among the minority that were opposed many left comments expressing strong opposition.

